# Positive Impact of ERAS Programme on Living and Deceased Donor Renal Transplant Recipients During COVID-19 Pandemic

**DOI:** 10.3389/ti.2025.14238

**Published:** 2025-08-21

**Authors:** Rachel A. B. Thomas, Hannah K. Chalmers, Helen M. E. Usher, Olivia Pestrin, Emily J. Simpson-Dent, Maia I. Webb, Hilary M. Guthrie, Sonia J. Wakelin, Gabriel C. Oniscu

**Affiliations:** ^1^ Edinburgh Transplant Centre, Edinburgh, United Kingdom; ^2^ Division of Transplantation Surgery, CLINTEC, Karolinska Institutet, Stockholm, Sweden

**Keywords:** enhanced recovery after surgery (ERAS), kidney, COVID-19, length of stay, perioperative care

Dear Editors,

We watch with interest as the transplant community adopts Enhanced Recovery After Surgery (ERAS) protocols. There was a significant reduction in recipient length of stay after introduction of an ERAS protocol in our unit when compared with an earlier, matched population with no evidence of adverse events and high acceptability to patients and staff. We believe that the ERAS protocol contributed to this difference and helped change staff mindset.

ERAS protocols are multi-modal perioperative care pathways designed to improve recovery after major surgery by maintaining preoperative body composition and physiological organ function and modifying the surgical stress response. ERAS is now standard of care in many surgical specialties as it reduces post-operative complications, pain, and length of stay (LoS) with increased patient satisfaction. ERAS in solid organ transplantation is less well established but there is increasing evidence that ERAS can positively impact on living donor, recipient and organ outcomes with additional financial benefits [1–7]. However, in a survey of all UK renal transplant units, only three had established ERAS programmes [3]. The most cited barrier was embedded peri-transplant management culture within the unit along with limited evidence and lack of existing guidelines in the heterogeneous UK transplant environment [1].

Our centre proposed an ERAS quality improvement programme before the COVID-19 pandemic, which accelerated momentum due to an increasing awareness of nosocomial transmission risk aiming to reduce hospital exposure for vulnerable transplant recipients. The aim of the ERAS quality improvement project was to create a straight-forward, acceptable, protocol that would result in a reduced in-patient stay aiming for day five discharge (as seen safely in other units [2, 4]) without an increase in readmissions or morbidity. A successful ERAS programme requires staff buy-in, managerial support and compliance to an agreed plan, so a multidisciplinary team meeting was convened where available literature, feedback from other UK units and patients were considered and a renal transplant recipient protocol agreed (Figure 1).

**FIGURE 1:**
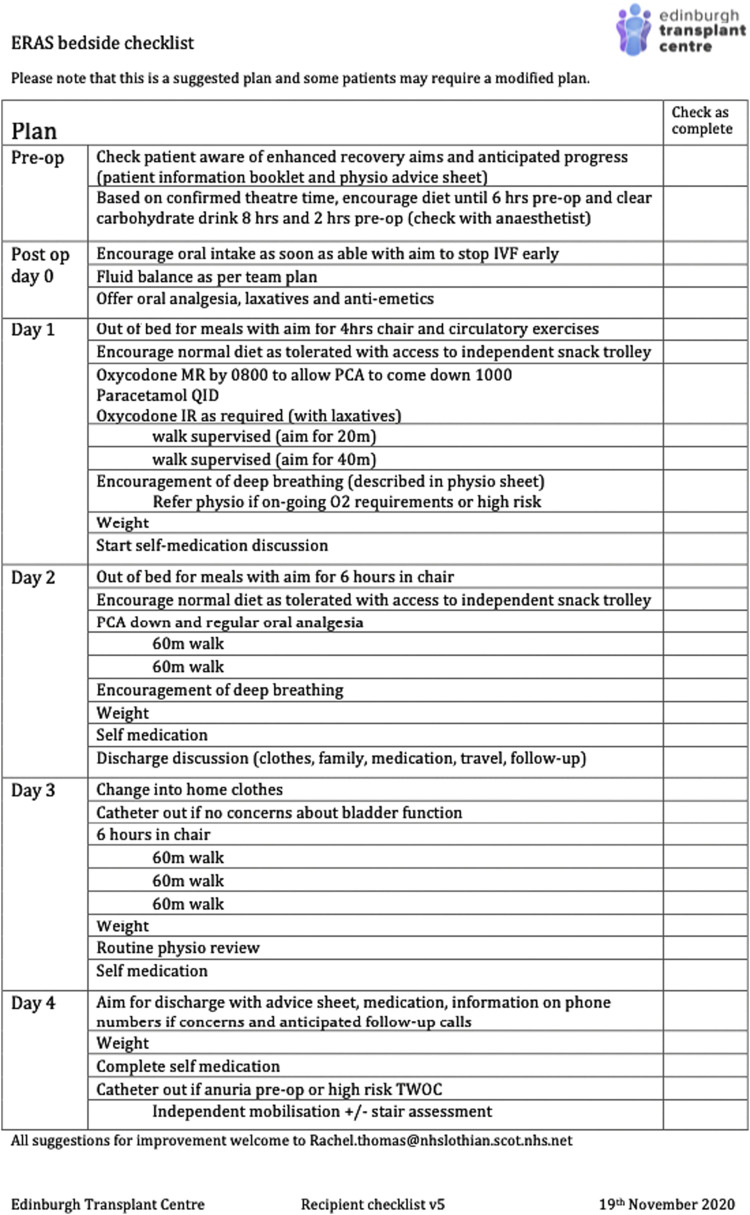
ERAS checklist.

The main differences included earlier catheter removal (day 3 as supported by Cole et al [7]), encouragement of early mobilisation goals through a new physiotherapy referral pathway, dietary advice and analgesia guidance to reduce opioid use. To further encourage mobilisation and reduce analgesia requirements, we recommended reduction of auxiliary lines including surgical drains, central lines (given that CVP is a poor marker for vascular filling in most cases [8]) and early discontinuation of IV fluids. No specific fluid overload scoring system has been validated and therefore we continued with individualised goal-directed fluid regimes to limit weight gain and reduce fluid overload that has negative impact on gut and graft function [1, 8].

Patients on the renal transplant waiting list and scheduled live donor transplant recipients were informed, and the updated recovery pathway was included in the pre-listing information briefing. Staff were updated through unit meetings and a local ERAS nurse delivered educational events to help with a smooth transition.

The outcomes from the first 35 consecutive renal transplants after introduction of ERAS (ERAS cohort, December 2020- February 2021) were compared to 35 consecutive recipients before the introduction (pre-ERAS cohort, June- November 2020). It is acknowledged that this comparison might be biased as ERAS had been discussed widely prior to implementation and our unit was already aiming for expedited discharge due to the pandemic. Therefore, an additional analysis of 35 consecutive pre-COVID-19 recipients were used for further LoS analysis (pre-COVID-19 December 2019-March 2020).

The median LoS for the 35 consecutive recipients on the ERAS pathway was 5 days, compared to 8 days (pre-COVID-19, p = 0.008) and 7 days (pre-ERAS or standard cohort p = 0.050) (Mann Whitney U test). The aim for day five discharge was met for 54% (19 patients) of the transplants compared to 17% pre-ERAS (X^2^, p = <0.001) and only two pre-COVID-19 (5%, X^2^, p = <0.001).

There was no evidence that the reduced LoS resulted in more readmissions within 30 days compared to either group (2 ERAS vs. 2 pre-ERAS vs. 7 pre-COVID-19) or more adverse events such as recatheterisation (1 vs. 4 vs. 3) or significant complications such as return to theatre (1 vs. 2 vs. 3).

There was no evidence that recipients in the ERAS group were less complex; they were older (median age 58 vs. 52 pre-ERAS vs. 53 pre-COVID-19, p = NS), had fewer live donor transplants (9 vs. 13 vs. 12, p = NS), less pre-emptive transplantation (5 vs. 9 vs. 7, p = NS) and were more obese (19 vs. 11 vs. 8 [BMI >30], p = 0.02). However, likely in keeping with the change in donor acceptance during COVID, there were equivalent DCD donors compared to pre-ERAS but more in pre-COVID-19 group (23% deceased donors vs. 21% vs. 39% but did not reach significance) and this may have impacted on LoS.

80% of ERAS patients met the goal of mobilisation on day one and were more likely to be discharged earlier from physio (median discharge day 3.6 ERAS vs. day 5.8 pre-ERAS). There was no significant difference in opiate use although a trend to less use (238 mg equivalent oral morphine dose vs. 266mg pre-ERAS). Unfortunately, two potential local analgesic methods (wound catheters and transversus abdominis plane (TAP) blocks), which can reduce opioid use and improve bowel and pulmonary function were not consistently available during this period [9].

Further analysis of those who did not meet the discharge target found that while some patients had graft issues that required longer inpatient stay for additional investigations or treatment, some (17%) could have been discharged earlier due to preventable issues such as medication issuing delay, earlier specialty referral and transport issues. This provided a focus for future improvements and recognises the need for system-wide buy-in to address all aspects of care. Of note, the lead author (and ERAS contact surgeon) left the unit and LoS was noted to have increased again to pre-ERAS levels (November 2022- February 23, median 8.5 days). While this increase is likely multifactorial, when reviewed, the ERAS checklist (Figure 1) was neither physically in the casenotes nor mentioned in electronic record for 70% (14/20) recipients, potentially underscoring the acknowledged benefit of a dedicated ERAS lead to drive the process, which is a challenge in an over-stretched health system.

Patients and staff satisfaction with ERAS was sought. All 35 ERAS patients were phoned for feedback and 56% responded. All who answered thought that their discharge was safe, had adequate analgesia and found physiotherapy input was helpful. 25/28 members of the MDT completed the written questionnaire with positive comments on analgesia, physio input and safety. 92% of staff and patients who completed the feedback recommended a continuation with the protocol in its current form.

We should also acknowledge that prehabilitation complements ERAS care and we aim to develop a prehabilitation programme, which may be funded by the savings associated with reduced LoS.

In conclusion, our implementation of ERAS for renal transplant was safe, feasible and acceptable, in line with results from other centres. We acknowledge that the reduction in length of stay may have been a multimodal response to practice change but the results from this retrospective, quality improvement project has encouraged development of pancreas and liver transplant recipient protocols and supported funding for a dedicated ERAS transplant nurse. ERAS guidelines for renal transplant are underway from the ERAS Society and NHS Blood and Transplant [10].

## Data Availability

The original contributions presented in the study are included in the article/supplementary material, further inquiries can be directed to the corresponding author.

## References

[1] Morkane CM Fabes J Banga NR Berry PD Kirwan CJ. Perioperative Management of Adult Cadaveric and Live Donor Renal Transplantation in the UK: A Survey of National Practice. Clin Kid J (2019) 12:880–7. 10.1093/ckj/sfz017 PMC6885684 31807303

[2] O’Neill S McGrogan D Sweeney N McDaid J Beckett N Magowan H Application of Enhanced Recovery After Surgery in Patients Undergoing Kidney Transplant: The Belfast Protocol. T Trans Proc (2021) 000:2204–5. 10.1016/j.transproceed.2021.07.046 34456045

[3] Amer A Scuffell C Dowen F Wilson CH Manas DM. A National Survey on Enhanced Recovery for Renal Transplant Recipients: Current Practices and Trends in the UK. Ann R Coll Surg Engl (2022) 000:1–7. 10.1308/rcann.2021.0365 PMC9889185 35446720

[4] Espino KA Narvaez JRF Ott MC Kayler LK. Benefits of Multimodal Enhanced Recovery Pathway in Patients Undergoing Kidney Transplantation. Clin Transpl (2018) 32(2):e13173. 10.1111/ctr.13173 29220082

[5] Halawa A Rowe S Robert F Nathan C Hassan A Kumar A A Better Journey for Patients, a Better Deal for the NHS: The Successful Implementation of an Enhanced Recovery Program After Renal Transplant Surgery. Exp Clin Transpl (2018) 2:127–33. 10.6002/ect.2016.0304 28836932

[6] Hanson N Peramunage D Kuhr CS Brandenberger J Cowan NG Flaherty JM Reduced Length of Hospitalization and Associated Healthcare Costs Using an Enhanced Recovery Pathway After Kidney Transplant Surgery. J Clin Anes (2020) 65:109855. 10.1016/j.jclinane.2020.109855 32413814

[7] Cole T Hakim J Shapiro R Kayler LK. Early Urethral (Foley) Catheter Removal Positively Affects Length of Stay After Renal Transplantation. Transplantation (2007) 83:995–6. 10.1097/01.tp.0000259723.92943.8f 17460573

[8] Campos L Parada B Furriel F Castelo D Moreira P Mota A. Do Intraoperative Hemodynamic Factors of the Recipient Influence Renal Graft Function. Transpl Proc (2012) 44:1800–3. 10.1016/j.transproceed.2012.05.042 22841277

[9] Chadha R Pai S Aniskevich S McClain R Egan B Webb C Nonopioid Modalities for Acute Postoperative Pain in Abdominal Transplant Recipients. Transplantation (2020) 104(4):694–9. 10.1097/TP.0000000000003053 31815897

[10] Tan JHS Bhatia K Sharma V Swamy M van Dellen D Dhanda R Enhanced Recovery After Surgery Recommendations for Renal Transplantation: Guidelines. BJS (2023) 110:57–9. 10.1093/bjs/znac325 36168725

